# Clinical Characteristics and Contemporary Prognosis of Ventricular Septal Rupture Complicating Acute Myocardial Infarction: A Single-Center Experience

**DOI:** 10.3389/fcvm.2021.679148

**Published:** 2021-09-13

**Authors:** Lu Wang, Li-Li Xiao, Chao Liu, Yan-Zhou Zhang, Xiao-Yan Zhao, Ling Li, Xiao-Fang Wang, Jian-Zeng Dong

**Affiliations:** ^1^Department of Cardiology, The First Affiliated Hospital of Zhengzhou University, Zhengzhou, China; ^2^Department of Cardiovascular Surgery, The First Affiliated Hospital of Zhengzhou University, Zhengzhou, China; ^3^Department of Cardiology, Beijing Anzhen Hospital, Capital Medical University, Beijing, China

**Keywords:** acute myocardial infarction, percutaneous transcatheter closure, surgery, prognosis, ventricular septal rupture (VSR)

## Abstract

**Objectives:** Ventricular septal rupture (VSR) is a rare but lethal complication of acute myocardial infarction (AMI). We conducted a retrospective analysis of the clinical characteristics of VSR patients and explored the risk factors for long-term mortality.

**Methods:** In this single-center cohort study, 127 patients diagnosed with post-AMI VSR between May 2012 and April 2019 were included. Demographic, clinical, operative, and outcome data were collected. The 30-day and long-term mortality were outcomes of interest. Cox proportional hazard regression analysis was used to explore the predictors of long-term mortality.

**Results:** The mean age of the VSR cohort was 66.6 ± 8.7 years, 67 (52.8%) were males. Among the 127 patients, 78 patients (61.4%) were medically managed, 31 (24.4%) patients underwent percutaneous transcatheter closure (TCC), and 18 (14.2%) patients received surgical repair. The median follow-up time was 1129 days [interquartile range: 802–2019 days]. The 30-day mortality of the medically managed group, percutaneous TCC group, and surgical management group was 93.6, 22.6, and 11.1%, respectively; and the long-term mortality was 96.2, 25.8, and 22.2%, respectively. VSR repair treatment including surgical management (HR 0.01, 95% CI 0.001–0.09, *p* < 0.001) and percutaneous TCC (HR 0.09, 95% CI 0.03–0.26, *p* < 0.001) was associated with a better prognosis, and cardiogenic shock (CS) (HR 9.30, 95% CI 3.38–25.62, *p* < 0.001) was an independent risk factor of long-term mortality.

**Conclusions:** The prognosis of VSR patients without operative management remains poor, especially in those complicated with CS. Timely and improved surgery treatment is needed for better outcomes in VSR patients.

## Introduction

Ventricular septal rupture (VSR), a rare complication of acute myocardial infarction (AMI), remains one of the most challenging clinical problems to treat (1). Though the incidence of VSR has decreased to 0.2–0.5% with the advent of reperfusion strategies nowadays, (2, 3) the outcome of patients who develop VSR remains poor and appears almost unchanged over the last few decades (4). The mortality rates of patients with medical management alone were extremely high; therefore, a surgical closure is recommended to be the standard therapy by the current ST-elevation MI (STEMI) guidelines of the American College of Cardiology Foundation/American Heart Association (ACCF/AHA) and the European Society of Cardiology (ESC) (5, 6). In the real-world clinical practice, surgery procedures were often performed during the subacute and chronic periods (≥2 weeks after the initial detection of VSR) (7). However, the better outcome observed with delayed repair operation is also criticized as a representation of survival bias, as many patients were too sick to wait for delayed surgery and died during the waiting period (8). Recently, percutaneous transcatheter closure (TCC) has emerged as an alternative to surgical closure of VSR but is mainly restricted to selected cases in which patients have small VSR in the subacute or chronic phase (9–11). Besides, some surgical modifications have been proposed but appear less promising due to the lack of the repeatability and effectiveness confirmed by other researchers (12–15). Current treatments for VSR vary greatly, but the results remain disappointing.

Data on VSR complicating AMI are limited in China. This study aimed to review the treatment of VSR complicating AMI in our single-center. We attempted to identify the current status, compare the treatment outcomes, explore the prognostic risk factors of VSR, and provide some information regarding the management of such patients.

## Methods

The present study was a single-center analysis of post-AMI VSR patients at the First Affiliated Hospital of Zhengzhou University, Henan, China. Patients who were admitted because of VSR complicating AMI were retrospectively enrolled between May 2012 and April 2019. The VSR was defined as a disruption in the ventricular septum with evidence of left-to-right shunt and was confirmed by transthoracic echocardiography (TTE) examination. The enrolled cases, which exclude VSR secondary to the presence of congenital heart disease or resulting from a previous surgical procedure or by trauma or other reasons. The study was approved by the Human Research Ethics Committee of the First Affiliated Hospital of Zhengzhou University and was performed in accordance with the Declaration of Helsinki (Approved No. of ethic committee SS-2019-001). Written informed consent was obtained from each participant at their enrollment.

To assess the baseline clinical characteristics of the study cohort, we retrospectively collected data concerning patients' demographics information, hemodynamic conditions, morbidities, echocardiographic features, coronary angiography (CAG) findings, physiological data, laboratory tests. The definition of each variable was in line with the cardiovascular data standards (16). The location of the rupture was defined as apical, anterior, or posterior. Cardiogenic shock (CS) definition was according to clinical and hemodynamic criteria, including hypotension [systolic blood pressure (SBP) <90 mm Hg for 30 min or need for supportive measures to maintain the systolic blood pressure of >90 mm Hg) and evidence of end-organ hypoperfusion (17). Information on postoperative complications in patients who received VSR repair was also recorded, including low cardiac output syndrome (LCOS), renal failure requiring renal replacement therapy (CRRT), residual shunt, hemolysis requiring blood transfusion, and the length of stay in the intensive care unit.

### Patient Treatment and Operation Management

All patients received standard therapy of AMI as clinically indicated. Once VSR diagnosis was established, patients were under close monitoring of hemodynamic status, consistent urine output, creatinine level, liver enzymes, and blood lactate concentration. At the same time, they were administered volume expansion, vasopressors, and inotropes, with additional therapy for preventing or treating multi-organ dysfunction syndrome. If the diagnosis of VSR is made prior to revascularization therapy, including the primary percutaneous coronary intervention (PCI) or coronary artery bypass grafting (CABG), prompt restoration coronary flow would be recommended for all patients. In our experience with the management of VSR patients, the surgical repair would be performed in the following cases: evidence of CS, patients with poor perfusion and cardiac output, signs of congestive heart failure, maximum use of vasopressors or intra-aortic balloon pumping (IABP) could not maintain the SBP, and evidence of end-organ hypoperfusion. However, there were a considerable number of VSR patients deteriorated rapidly in the study, which making them unable to receive surgical/percutaneous intervention and leading to high mortality among medically treated patients.

Since there is no consensus on the optimal timing for surgery nowadays, the first 2 weeks of VSR onset were defined as the acute phase. Thus, the acute or early VSR closure means that the VSR repair operation, including the percutaneous TCC and surgery, was performed in the first 2 weeks after VSR onset. A delayed elective repair approach 3–4 weeks later may be considered in patients who respond well to aggressive heart failure therapy, including medication treatment or IABP. Besides, hemodynamically stable patients with a size <15 mm apical located VSR or with residual VSR after the initial surgical approach might be suited to receive percutaneous TCC repair procedure after the acute phase (9). If a delayed elective repair strategy were chosen, the coronary flow would be restored in the infarct-related artery with aspiration thrombectomy and/or balloon angioplasty, or PCI procedure with bare-metal stent placement, or preparations made for VSR repair with CABG. The use of mechanical circulatory or ventilatory support, as well as the proper VSR repair strategy, and the choice of revascularization therapy, were left to the consensus of experienced cardiologists and cardiac surgeons, and the final decision was made by the patient and his/her family.

### Follow-Up and Outcomes

The outcomes were the 30-day mortality and the long-term mortality. The Long-term mortality was defined as overall mortality during the follow-up period. The long-term outcomes were obtained by contacting each patient individually with monthly telephone interviews by trained staff.

### Statistical Analysis

Continuous variables were presented as the mean and standard deviation or median [Interquartile range (IQR)] and compared with one-way analysis of variance (ANOVA) or the Mann-Whitney or Kruskal-Wallis test. Categorical variables were expressed as frequency (percentage) and assessed by the Chi-square test or Fisher's exact test (when at least an expected value in a cell is <5). Cumulative incidence rates of unadjusted long-term mortality in patients with different management were estimated by the Kaplan-Meier method and compared with the log-rank test. Cox proportional hazard regression was performed to evaluate the HR and 95% CI for the association between risk factors and long-term mortality. The factors entered into the regression analysis were as follows: management of VSR, revascularization therapy, age, sex, CS, VSR type, size of main VSR, VSR location, complicated with ventricular arrhythmia, IABP support, previous histories of MI, hypertension, diabetes, heart rate, SBP, left ventricular eject fraction, white blood cell, estimated glomerular filtration rate, N-terminal pro b-type natriuretic peptide (NT-proBNP), cardiac troponin I (CTNI), creatine kinase MB (CK-MB), lactate dehydrogenase (LDH), and aspartate transaminase (AST). Levels of NT-proBNP, CTNI, CK-MB, LDH, and AST were normalized by log_10_ transformation. The assumption of proportional hazards was assessed with time-dependent covariate test methods, and none of the covariates was time-dependent (*P* > 0.05) (Online source 2). All analyses were performed using R (The R version 4.0.3; http://www.r-project.org). A 2-sided *p* < 0.05 was considered to be statistically significant.

## Results

Between May 2012 and April 2019, a total of 127 patients with a diagnosis of VSR complicating AMI were consecutively enrolled in this analysis. The present VSR cohort had a mean age of 66.6 ± 8.7 years, 67 (52.8%) were males. Among the 127 VSR patients, 78 (61.4%) patients were medically managed which meant treated conservatively, 31 (24.4%) patients went through percutaneous TCC, and 18 (14.2%) patients underwent surgical repair. Baseline characteristics according to patients' management strategies were detailed in Table 1. Patients in the conservatively treated group were more likely to be older, have acute VSR type, have a higher prevalence of CS (63/78, 80.8%), and a higher level of NT-proBNP and CK-MB, but more likely to have a smaller VSR size.

**Table 1:** Characteristics of VSR patients stratified by different managements.

**Characteristics**	**Medical management (without VSR repair) (*n* = 78)**	**Percutaneous TCC management (*n* = 31)**	**Surgical management (*n* = 18)**	***P* value**
Age, (years)	68.5 ± 8.7	64.0 ± 8.96	63.1 ± 5.53	**0.008**
Male sex, *n* (%)	36 (46.2)	19 (61.3)	12 (66.7)	0.162
Cardiogenic shock, *n* (%)	63 (80.8)	4 (12.9)	9 (50.0)	** <0.001**
AMI to VSR time, (days)	3.2 ± 2.4	3.8 ± 2.8	3.5 ± 2.6	0.448
^*^VSR Type, *n* (%)				0.400
Acute	28 (36.4)	8 (25.8)	5 (27.8)	
Subacute	22 (28.6)	8 (25.8)	6 (33.3)	
Late presentation	27 (35.1)	15 (48.4)	7 (38.9)	
Size of main VSR, (mm)	9.3 ± 5.3	10.9 ± 5.01	15.7 ± 6.16	** <0.001**
Single VSR, *n* (%)	56 (82.4)	26 (83.9)	17 (94.4)	0.449
VSR location, *n* (%)				0.332
Apical	62 (79.5)	20 (64.5)	11 (66.7)	
Anterior	9 (11.5)	6 (19.4)	3 (16.7)	
Posterior	7 (9.0)	5 (16.1)	4 (22.2)	
VSR to operation time (days)	–	20.0 [14.0–27.0]	14.0 [11.75–20.25]	0.018
Acute phase repair, *n* (%)	–	8 (25.8)	10 (55.6)	
Elective repair, *n* (%)		23 (74.2)	8 (44.4)	
MI information	–		
Infarct territory, n (%)				0.953
Anterior	63 (81.8)	25 (80.6)	14 (77.8)	
Inferior	11 (14.3)	5 (16.1)	4 (22.2)	
Others	3 (3.9)	1 (3.2)	–	
Prior Fibrinolysis therapy, *n* (%)	13 (16.7)	2 (6.5)	3 (16.7)	0.369
CAG data, *n* (%)				** <0.001**
Negative	1 (5.3)	3 (9.7)	1 (5.6)	
LAD	16 (84.2)	20 (64.5)	10 (55.6)	
RCA	2 (10.5)	4 (12.9)	3 (16.7)	
LCX	–	1 (3.2)	–	
Triple vessel disease	–	2 (6.5)	2 (11.1)	
Culprit artery treatment, *n* (%)				** <0.001**
PCI	16 (79.5)	15 (48.4)	4 (22.2)	
CABG	–	1 (3.2)	9 (50.0)	
PCI+CABG	–	1 (3.2)	2 (11.1)	
In-hospital stays, (days)	5.0 [2.0–11.0]	27.0 [19.0–32.0]	31.5 [22.7–40.3]	** <0.001**
**Comorbidities**, ***n*****(%)**				
Current smoker	16 (20.5)	11 (35.5)	5 (27.8)	0.260
Current drinker	11 (14.1)	5 (16.1)	3 (16.7)	0.942
Hypertension	42 (53.8)	16 (51.6)	11 (61.1)	0.807
Diabetes mellitus	25 (32.1)	7 (22.6)	7 (38.9)	0.453
History of MI	5 (6.4)	1 (3.2)	2 (11.1)	0.550
History of stroke/TIA	16 (20.5)	1 (3.2)	4 (22.2)	0.072
Hyperlipidemia	8 (10.3)	2 (6.5)	3 (16.7)	0.526
**Examinations**				
Heart rate, (b.p.m.)	92.6 ± 19.6	92.1 ± 15.4	93.2 ± 15.3	0.977
SBP, (mmHg)	105.9 ± 18.3	108.5 ± 14.7	114.3 ± 16.5	0.176
DBP, (mmHg)	68.9 ± 15.7	71.1 ± 9.8	74.1 ± 11.4	0.344
LVEF, (%)	49.6 ± 10.1	52.7 ± 9.3	52.2 ± 8.9	0.299
NT-pro BNP, (pg/mL)	9517.5 [4613.8–18367.7]	5160.0 [2610.5–10032.9]	5861.0 [2593.8–8693.8]	**0.013**
CK-MB, (mmol/L)	35.0 [15.5–74.9]	16.0 [9.5–24.1]	19.0 [15.5–29.6]	**0.003**
CTnI, (mmol/L)	2.0 [0.96–5.24]	0.74 [0.22–3.99]	0.22 [0.05–8.79]	0.134
LDH, (U/L)	737.0 [399.0–1229.0]	604.0 [361.0–745.0]	460.5 [288.8–915.5]	0.248
AST, (mmol/L)	84.0 [33.5–328.5]	37.0 [21.0–58.5]	31.0 [20.0–278.0]	0.191
Hemoglobin, (g/L)	121.4 ± 15.8	129.1 ± 47.3	126.3 ± 19.8	0.413
WBC, (10^3^/μL)	13.0 ± 6.43	10.9 ± 6.18	11.8 ± 4.26	0.277
Creatinine, (μmol/L)	101.5 [74.7–159.2]	88.4 [78.5–100.4]	94.7 [78.3–137.5]	0.328
Blood urea nitrogen, (mmol/L)	12.2 ± 8.74	9.40 ± 4.58	12.0 ± 8.19	0.252
eGFR, (mL/min/1.73 m^2^)	73.6 ± 34.2	68.5 ± 18.9	61.8 ± 27.2	0.208
Total bilirubin, (mmol/L)	20.6 ± 16.8	16.4 ± 9.45	21.3 ± 18.0	0.426
Albumin, (mmol/L)	36.9 ± 9.75	37.1 ± 7.74	37.5 ± 6.82	0.965
LDL-C, (mmol/L)	2.25 ± 0.74	2.35 ± 0.96	2.32 ± 0.80	0.857
30-day mortality, *n* (%)	73 (93.6)	7 (22.6)	2 (11.1)	** <0.001**
Long-term mortality, *n* (%)	75 (96.2)	8 (25.8)	4 (22.2)	** <0.001**
Survival time (days)	5.0 [2.0–12.0]	892.0 [45.0–1698.0]	1059.0 [529.75–1323.25]	** <0.001**

*VSR, ventricular septal rupture; TCC, Percutaneous transcatheter closure;*

* *VSR Type: Acute (Within 24 hours Post AMI), Subacute (Within 24–72 h Post AMI), Late presentation (More than 72 h Post AMI); SD, Standard Deviation; AMI, acute myocardial infarction; IABP, intra-aortic balloon pump; ECMO, extracorporeal membrane oxygenation; STEMI, ST segment elevate myocardial infarction; CAG, coronary angiography; LAD, left anterior descending; RCA, right coronary artery; LCX, left circumflex coronary artery; PCI, percutaneous coronary intervention; CABG, coronary artery bypass surgery; TIA, transient ischemic attack; SBP, systolic blood pressure; DBP, diastolic blood pressure; LVEF, left ventricular eject fraction; NT-pro BNP, N-terminal pro b-type natriuretic peptide; CK-MB, creatine kinase MB; CTNI, cardiac troponin I; LDH, lactate dehydrogenase; WBC, white blood cell; eGFR, estimated glomerular filtration rate; AST, aspartate transaminase; LDL-C, low-density lipoprotein cholesterol. P values in bold meant significantly different (P < 0.05)*.

The 30-day mortality rate of the conservatively treated group, percutaneous TCC group, and surgical management group was 93.6% (73/78), 22.6% (7/31), and 11.1% (2/11), respectively. The median duration of follow-up was 1129 days [Interquartile range (IQR): 802–2019 days]. During the follow-up period, another five patients (2 in the medical management group, 1 in the percutaneous TCC group, and 2 in the surgical management group) died, and no patient was lost to follow-up. The main complications included CS (76, 59.8%), ventricular arrhythmia (16, 12.6%), ventricular aneurysm (75, 59.5%), and use of IABP (21, 16.5%). Causes of death included LCOS (32, 25.2%), refractory heart failure (46, 36.2%), multiple-system organ failure (5, 3.14%), hemorrhage event (2, 1.57%), and unknown reasons (2, 1.57%) (Table 2).

**Table 2:** Clinical complications, causes of death, and outcomes of patients with VSR.

**Clinical complications/outcomes**	**Patients *N* = 127**
30-day mortality, *n* (%)	82 (64.6)
Overall mortality, *n* (%)	87 (68.5)
Survival time (days)	15.0 [3.0–784.0]
Cardiogenic shock, *n* (%)	76 (59.8)
Ventricular arrhythmia, *n* (%)	16 (12.6)
Ventricular aneurysm, *n* (%)	75 (59.5)
IABP support, *n* (%)	21 (16.5)
**Causes of death**	**Patients** ***N*** **=** **127**
Low cardiac output syndrome, *n* (%)	32 (25.2)
Refractory heart failure, *n* (%)	46 (36.2)
Multiple-system organ failure, *n* (%)	5 (3.14)
Hemorrhage event, *n* (%)	2 (1.57)
Unknown reasons, *n* (%)	2 (1.57)
**Peri-operative complications**	**Patients** ***N*** **=** **49**
Surgical management, *n* (%)	31 (63.3)
Percutaneous TCC, *n* (%)	18 (36.7)
Operation failure, *n* (%)	3 (6.12)
Postoperative IABP, *n* (%)	8 (16.3)
Postoperative ECMO, *n* (%)	2 (4.08)
Postoperative CRRT, *n* (%)	4 (8.16)
Residual VSR, *n* (%)	15 (30.6)
Postoperative hemolysis, *n* (%)	3 (6.12)

*VSR, ventricular septal rupture; TCC, Percutaneous transcatheter closure; IABP, intra-aortic balloon pump; ECMO, Extracorporeal membrane oxygenation; CRRT, continuous renal replacement therapy*.

As for the 31 patients who received percutaneous TCC repair procedures, two operations failed. The first failure resulted from an appropriate position that could not be found for releasing the closure device. In another patient, the failure was due to the inability to fix the closure device. Among patients with surgical repair operations, ten patients were repaired with David's infarction exclusion technique (18). One of ten cases failed for the rupture was too large to be closed, and the patient died one day after the surgery procedure. The remaining eight patients received a modified surgery method named SurCOP (Surgical repair Combining an Occluder and a Patch) with a 100% success rate. SurCOP repair technique upgraded the VSR repair material from a simple patch to a patch combined with an arterial catheter occluder which was first performed in our institution and associated with promising results for prognosis (19). The postoperative complications were: use of IABP (8, 16.53%), use of ECMO (2, 4.08%), use of CRRT (4, 8.16%), residual VSR (15, 30.6%), and hemolysis (3, 6.12%). Patients with VSR repair management according to operation timing were also compared, and patients who receive an early VSR repair operation had higher mortality than those who underwent the delayed surgery. Detailed individual patient data and outcomes with operative management were shown in Supplementary Tables 1–3.

**Figure 1:**
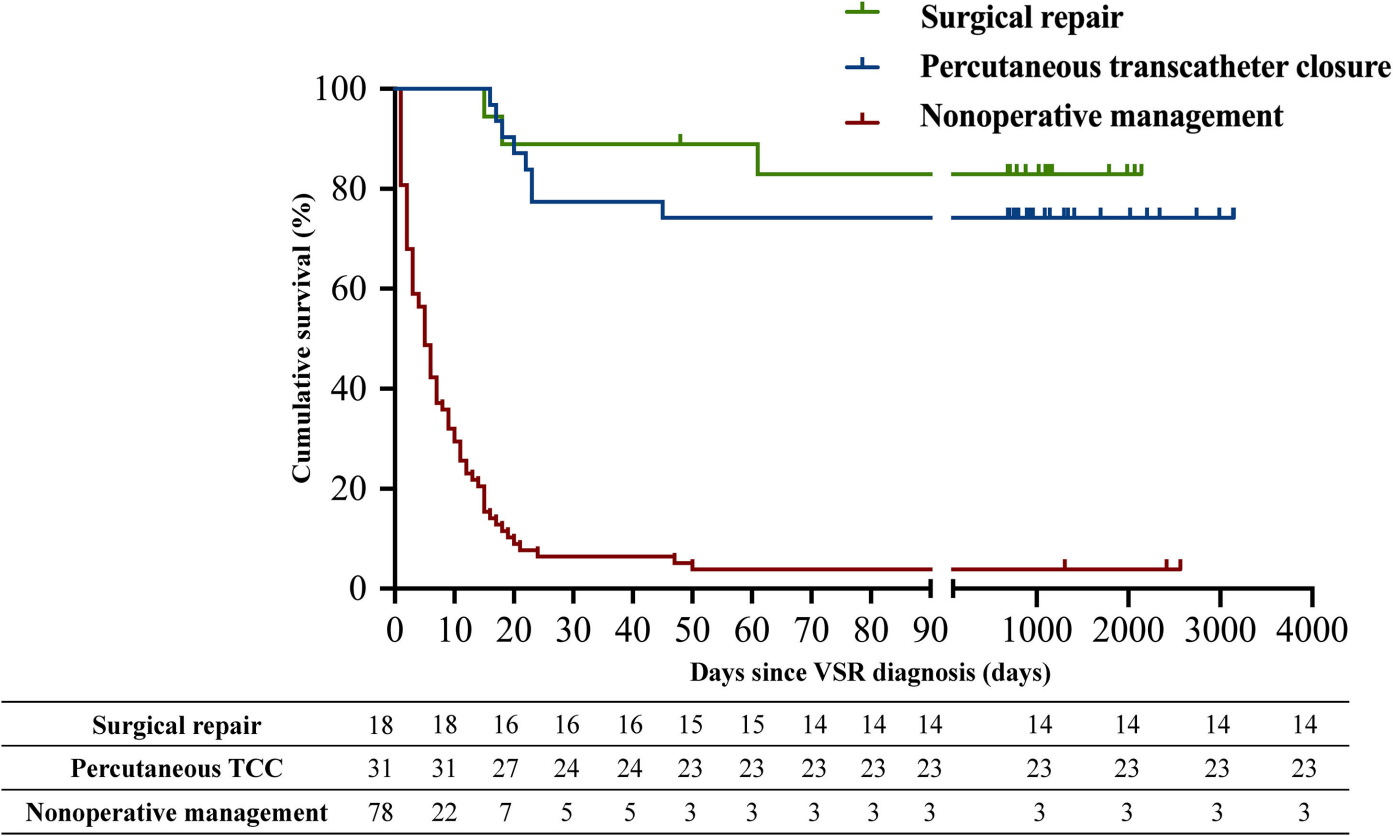
Kaplan-Meier estimates of cumulative event-free survival for long-term mortality.

Figure 1 showed the difference in the cumulative long-term survival rates of patients with different management strategies. Patients with medical management had a significantly higher long-term mortality compared with those received percutaneous TCC and surgical management [Medical management (96.2%, 75/78) vs. percutaneous TCC (25.8%, 8/31), *p* < 0.001; Medical management vs. surgery (22.2%, 4/18), *p* < 0.001]. There was no significant survival difference between the percutaneous TCC and surgical management group (*p* = 0.742). Survival analysis using the Cox regression model showed that VSR medical management, non-revascularization therapy, advanced age, concomitant CS, acute VSR type, complicated with ventricular arrhythmia, lower systolic blood pressure and left ventricular eject fraction, higher level of log NT-proBNP, log CTNI, log CK-MB, log LDH, log AST, white blood cell count, and estimated glomerular filtration rate <60 mL/min/1.73 m^2^ were univariate predictors of the long-term mortality. Moreover, the multivariate Cox regression analysis revealed that VSR repair treatment including percutaneous TCC (HR 0.09, 95% CI 0.03–0.26, *p* < 0.001) and surgical management (HR 0.01, 95% CI 0.001–0.09, *p* < 0.001) was associated with a better long-term prognosis, and CS (HR 9.30, 95% CI 3.38–25.62, *p* < 0.001) was associated with a poor future outcome (Table 3).

**Table 3:** Independent risk factors of long-term mortality.

**Predictors**	**Long-term mortality**					
	**Unadjusted HR**	**95% CI**	***P* value**	**Adjusted HR**	**95% CI**	***P* value**
Management of VSR (Medical management as reference)			<0.001			<0.001
Percutaneous transcatheter closure	0.078	0.037–0.167	<0.001	0.09	0.03–0.26	<0.001
Surgery	0.066	0.023–0.183	<0.001	0.01	0.001–0.09	<0.001
Revascularization therapy (Medical management as reference)			0.001	0.90	0.40–2.04	0.802
PCI	0.46	0.28–0.76	0.002			
CABG	0.11	0.03–0.47	0.003			
Age (year)	1.03	1.01–1.06	0.008	0.99	0.95–1.03	0.588
Male sex	0.67	0.44–1.02	0.064	1.07	0.46–2.51	0.873
Cardiogenic shock	5.39	3.23–8.99	<0.001	9.30	3.38–25.62	<0.001
VSR type (Acute VSR as reference)			0.048			0.277
Subacute	0.77	0.46–1.29	0.322	0.64	0.26–1.56	0.326
Late presentation	0.53	0.32–0.88	0.014	0.47	0.19–1.20	0.115
Size of main VSR	0.97	0.93–1.01	0.188	1.02	0.95–1.09	0.687
VSR Location (Apical as reference)			0.201			0.866
Anterior	0.77	0.42–1.43	0.410	0.71	0.20–2.51	0.597
Posterior	0.53	0.26–1.11	0.092	0.91	0.21–3.95	0.897
Complicated with ventricular arrhythmia	1.85	1.05–3.26	0.032	0.43	0.13–1.47	0.179
IABP support	1.05	0.65–1.71	0.843	0.67	0.28–1.57	0.353
Previous history of MI	0.79	0.32–1.94	0.603	1.36	0.24–7.64	0.727
Hypertension	0.98	0.65–1.50	0.933	1.03	0.45–2.35	0.954
Diabetes	1.20	0.77–1.87	0.419	1.15	0.53–2.50	0.725
Heart rate	1.01	0.99–1.02	0.081	1.01	0.98–1.03	0.600
Systolic blood pressure	0.98	0.97–0.99	0.015	0.99	0.97–1.01	0.363
Left ventricular eject fraction	0.98	0.95–0.99	0.033	0.99	0.96–1.04	0.959
Log NT-pro BNP	4.26	2.30–7.88	<0.001	1.74	0.47–6.39	0.406
Log CTNI	3.12	1.95–5.01	<0.001	1.27	0.70–2.31	0.472
Log CK-MB	1.51	1.19–1.91	0.001	1.75	0.47–6.39	0.436
Log LDH	2.58	1.30–5.09	0.006	1.54	0.21–11.1	0.667
Log AST	2.37	1.69–3.30	<0.001	0.67	0.26–1.70	0.397
WBC	1.05	1.02–1.08	0.002	1.04	0.98–1.11	0.228
eGFR (>90 mL/min/1.73 m^2^ as reference)			0.009			0.664
60–90 mL/min/1.73 m^2^	1.20	0.70–2.05	0.516	1.52	0.52–4.72	0.427
<60 mL/min/1.73 m^2^	2.22	1.30–3.80	0.004	1.57	0.57–4.04	0.401

*HR, hazard ratios; CI, confidence intervals; VSR, ventricular septal rupture; NT-pro BNP, N-terminal pro b-type natriuretic peptide; CTNI, cardiac troponin I; CK-MB, creatine kinase MB; LDH, lactate dehydrogenase; AST, aspartate transaminase; WBC, white blood cell; eGFR, estimated glomerular filtration rate*.

## Discussion

Despite improvements in medical treatment and revascularization techniques during the last two decades, the prognosis of post-AMI VSR remained disappointing. In this single-center retrospective cohort study, we found that the mortality of patients treated conservatively was extremely high, with a 30-day mortality rate of 93.6% (73/78) and a long-term mortality rate of 96.2% (75/78), which was consistent with previous studies (3, 4). Patients who survived the early stage and underwent VSR repair surgery showed a good long-term prognosis, either they received percutaneous TCC operation (25.8%, 8/31) or surgical repair (22.2%, 4/18). However, whether VSR is repaired or not, CS was found to be the independent predictor of poor prognosis, which multiplied the long-term mortality rate by nearly 9.3 times.

VSR results from full-thickness MI of the interventricular septum, leading to acute left-to-right shunting and superseding biventricular failure, CS, and finally, death (20). Previous studies have found that advanced age, (21) the shorter time between AMI and surgery, (22) posterior septal rupture, (23) incomplete coronary revascularization, (24) and right ventricular dysfunction (25) were independent predictors of mortality. However, this study found that all the above factors were pale and powerless if adjusted with CS. Compared to those without CS, patients with CS at the initial onset of VSR were 2–5 times more likely to die (26–28). In this study, CS was the main cause of death, and the high mortality rate of patients with conservative treatment was mainly due to the high prevalence rate of CS (80.8%, 63/78). Therefore, it is significant to stabilize patients' hemodynamics status in the early stage to improve their survival chances. Nowadays, in addition to the optimal use of afterload reducing agents, early use of mechanical circulatory support including IABP and ECMO can rapidly improve the hemodynamic status and provide a better condition for patients with CS to receive delayed surgery (29, 30). In the presented study, the application rate of preoperative IABP and ECMO was low, which might be part of the reason for the high early mortality rate. Directly closes the rupture site by surgery is the definitive treatment for patients with VSR, and it is associated with a promising prognosis (20). Our study found that VSR repair treatment, including surgical management (HR 0.01) and percutaneous TCC (HR 0.09), was associated with an improved long-term prognosis.

Although a surgical repair is highly recommended for patients with VSR, the appropriate timing of repair surgery remains elusive. Previous studies showed that the operative mortality was extremely high when the surgery was done urgently and decreased dramatically when it was intentionally delayed (31). The 2013 American College of Cardiology and American Heart Association guidelines recommended emergency surgical repair once the VSR was diagnosed, regardless of the hemodynamic status (5). Whereas, the 2017 European Society of Cardiology guidelines prefer delaying VSR repair operation in patients who respond well to aggressive medication therapy (6). Unfortunately, the better outcome of an elective surgery might be a manifestation of survival bias. After a waiting period of 4–6 weeks, the necrotic myocardium could undergo fibrotic remodeling, and the tensile strength is high enough to sustain the defect interpolation (32). This will definitely improve the prognosis of those patients who can survive the waiting period. However, those patients with hemodynamic instability could not survive long enough to receive the surgical intervention (7). Our research was no exception to this bias. In the present study, the median survival time of medically treated patients was 5 days, and more than 75% of patients died within 12 days of VSR symptom onset, but the median time from onset of VSR to the repair procedure was about 18.0 [13.0–24.5] days. As an early surgery intervention offers the only realistic chance of survival and each survivor is something to treasure, we cannot help thinking that these patients might derive the maximal benefit from much earlier and aggressive surgical therapy. Though surgical mortality of VSR patients with CS in the early phase remains very high, non-surgical mortality is undoubtedly higher. Thus, we clinicians should weigh the risk of the extended indications of repair surgery against the risk of postponing surgery and developing further clinical deterioration.

Nowadays, the David infarct exclusion (IE) strategy has been widely used in surgical procedures (18). In fact, insertion of a large patch that bears strong tension might tear off the friable myocardium from the suture line, resulting in the significant operative complication of concern, postoperative residual shunt, which has been considered the most crucial risk factor for poor outcomes (33). Percutaneous TCC has recently emerged as a potential strategy; however, it is mainly restricted to patients with a small VSR in the subacute or chronic phase. When operated in the acute phase, TCC is associated with high operative mortality (34). An improved technique of both surgical and percutaneous repairs was urgently needed. Recently, numerous modifications of the surgical techniques have been proposed: two patches with or without gelatin-resorcin-formalin, three-patch technique, local applications of adhesives, etc. (12–15). Regrettably, these techniques tend to be more complicated, and the reproducibility issue is of utmost concern. Based on our experience with the management of VSR, we also proposed a modified surgical repair technique, named SurCOP (Surgical repair Combining an Occluder and a Patch), which combines the use of a patent ductus arteriosus occluder with a slightly larger bovine pericardial patch to close the rupture site and the preliminary results have been reported (19). Till April 2019, the SurCOP was performed on eight patients, and our experience has shown that the SurCOP technique is a safe, easy-to-manipulate, and effective method that can be used in patients with hemodynamic instability.

The present study increased our knowledge of the current status of this rare complication and demonstrated the mortality risk factors of VSR. There are several other limitations to this investigation. First, the better results of a VSR repair surgery might be a manifestation of survival bias, as it is usually performed in relatively stable patients with VSR who are expected to have a better prognosis than those patients with hemodynamic instability in the early stage. Our research was no exception to this bias. Second, this study was retrospective, and patient selection could not be randomized. Although all data were collected retrospectively, selection and recall bias could not be completely prevented. Another consideration is that all patients in the research were enrolled from a single center; thus, their prognoses might be the results of more specialized teams with sufficient expertise instead of more general facilities.

## Conclusion

VSR is relatively rare but highly lethal in clinical practice. The outcomes of patients with VSR are still disappointing. The patient's survival chance depends on the intervention closure of the VSR. Thus, the coordination of surgical expertise and the application of novel treatment methods are required to improve the clinical outcomes of patients with VSR.

## Data Availability Statement

The raw data supporting the conclusions of this article will be made available by the authors, without undue reservation.

## Ethics Statement

The studies involving human participants were reviewed and approved by the Human Research Ethics Committee of the First Affiliated Hospital of Zhengzhou University. The patients/participants provided their written informed consent to participate in this study. Written informed consent was obtained from the individual(s) for the publication of any potentially identifiable images or data included in this article.

## Author Contributions

LW, X-FW, and J-ZD: study planning, study analysis, and responsible for the overall content as guarantors. CL, Y-ZZ, X-YZ, and LL: conducted the study and performed the examinations. LW and L-LX: performed the statistical analysis and wrote the manuscript. All authors have read and approved the final version of the manuscript.

## Conflict of Interest

The authors declare that the research was conducted in the absence of any commercial or financial relationships that could be construed as a potential conflict of interest.

## Publisher's Note

All claims expressed in this article are solely those of the authors and do not necessarily represent those of their affiliated organizations, or those of the publisher, the editors and the reviewers. Any product that may be evaluated in this article, or claim that may be made by its manufacturer, is not guaranteed or endorsed by the publisher.
