# Supplementary material for: Clinical Characteristics and Contemporary Prognosis of Ventricular Septal Rupture Complicating Acute Myocardial Infarction: A Single-Center Experience
Source: Front Cardiovasc Med. 2021 Sep 13;8:679148. doi: 10.3389/fcvm.2021.679148 (PMC8473686; doi:10.3389/fcvm.2021.679148)
Supplement: Supplementary file 1 [file Table_1.DOCX]

**Online Table 1: Individual characteristics of VSR patients receiving surgical management**

| **Patient No.** | **Operation**  **time** | **Age** | **Sex** | **Cardiac shock** | **AMI to VSR (days)** | **AMI to operation (days)** | **VSR to operation (days)** | **VSR location** | **Main VSR size** | **Culprit vessel** | **Reperfusion therapy** | **Surgery**  **Technique** | **Closure size** | **Complication, post-operation support** | **ICU stays**  **(days)** | **Alive** | **Cause of death** | **Survival**  **Days** |
| --- | --- | --- | --- | --- | --- | --- | --- | --- | --- | --- | --- | --- | --- | --- | --- | --- | --- | --- |
| **1** | 7-Nov-14 | 67 | F | Y | 1 | 8 | 7 | Apical | 8.5 | Triple vessel disease | PCI | IE |  | **Failure** | 2 | **N** | **LCOS** | 15 |
| **2** | 18-Feb-15 | 61 | M | N | 3 | 21 | 18 | Posterior | 18 | LAD | CABG | IE |  |  | - | Y |  | 2143 |
| **3** | 3-May-15 | 65 | F | N | 7 | 21 | 14 | Apical | 8 | LAD | PCI | IE |  | Ventricular aneurysm resection | - | Y |  | 2069 |
| **4** | 22-Jun-15 | 52 | F | N | 1 | 21 | 20 | Apical | 22 | - | CABG | IE |  |  | - | Y |  | 1986 |
| **5** | 7-Feb-16 | 65 | M | N | 1 | 28 | 27 | Apical | 7 | LAD | CABG | IE |  |  | - | Y |  | 1789 |
| **6** | 16-Aug-17 | 65 | M | Y | 3 | 15 | 12 | Apical | 20 | Triple vessel disease | - | SurCOP | 24/26 | Ventricular aneurysm resection, IABP, CRRT, ECMO | 19 | **N** | **Abdominal hemorrhage^*^** | 18 |
| **7** | 20-Oct-17 | 59 | M | N | 10 | 42 | 32 | Apical | 7 | - | PCI+CABG | IE |  |  | 2 | Y |  | 1168 |
| **8** | 28-Nov-17 | 60 | F | Y | 1 | 20 | 19 | Anterior | 14 | LAD | CABG | SurCOP | 20/22 |  | 19 | Y |  | 1129 |
| **9** | 5-Dec-17 | 72 | F | N | 4 | 25 | 21 | Apical | 12 | LAD | PCI | IE |  | Ventricular aneurysm resection, Tricuspid annuloplasty | - | Y |  | 1122 |
| **10** | 28-Dec-17 | 63 | F | N | 5 | 29 | 24 | Anterior | 20 | LAD | CABG | IE |  |  | - | Y |  | 1099 |
| **11** | 1-Jan-18 | 54 | M | Y | 2 | 17 | 15 | Apical | 20 | LAD | 0 | SurCOP | 18/20 | Ventricular aneurysm resection | 9 | Y |  | 1095 |
| **12** | 14-Mar-18 | 64 | M | Y | 2 | 14 | 12 | Posterior | 20 | RCA | CABG | SurCOP | 24/26 |  | 3 | Y |  | 1023 |
| **13** | 12-Jun-18 | 71 | M | Y | 5 | 14 | 9 | Anterior | 15 | LAD | CABG | SurCOP | 16/18 | Ventricular aneurysm resection, IABP, CRRT | 11 | **N** | **Hemorrhagic stroke^#^** | 48 |
| **14** | 3-Aug-18 | 68 | M | N | 1 | 14 | 13 | Apical | 20 | LAD | PCI+CABG | IE |  | Ventricular aneurysm resection | 12 | Y |  | 881 |
| **15** | 25-Sep-18 | 59 | M | N | 7 | 21 | 14 | Posterior | 30 | RCA | CABG | IE |  | CRRT | 8 | **N** | **LCOS** | 61 |
| **16** | 8-Nov-18 | 64 | M | Y | 3 | 14 | 11 | Apical | 11 | - | - | SurCOP | 24/26 | Ventricular aneurysm resection | 6 | Y |  | 784 |
| **17** | 23-Jan-19 | 69 | M | Y | 5 | 14 | 9 | Apical | 15 | LAD | PCI | SurCOP | 20/22 | Tricuspid annuloplasty | 21 | Y |  | 708 |
| **18** | 14-Feb-19 | 57 | M | Y | 2 | 14 | 12 | Posterior | 15 | RCA | PCI+CABG | SurCOP | 20/22 | IABP | - | Y |  | 686 |

AMI(acute myocardial infarction),VSR (ventricular septal rupture), ICU (intensive care unit), Y (yes), N (no), M(male), F(female), IE(infarction exclusion strategy), SurCOP (surgical repair combining Patent ductus arteriosus occluder and patch), LAD (left anterior descending coronary), RCA (right coronary artery), PCI (percutaneous coronary intervention), CABG (coronary artery bypass grafting), IABP (intra-aortic balloon pump), CRRT (continuous renal replacement therapy), ECMO (Extracorporeal membrane oxygenation), LCOS (Low cardiac output syndrome), Abdominal hemorrhage**^*^**: confirmed by laparotomy (might be a complication of operation); Hemorrhagic stroke**^#^**: confirmed by a head computer tomography examination (might related to the anti-platelet therapy). Survival time were calculated from the day after VSR operation till the last follow-up (31-Dec-2020)

**Online Table 2: Individual characteristics of VSR patients receiving percutaneous TCC**

| **Patient No.** | **Operation time** | **Age** | **Sex** | **Cardiac shock** | **AMI to VSR**  **(days)** | **AMI to operation (days)** | **VSR to operation (days)** | **VSR location** | **Main VSR size** | **Culprit vessel** | **Reperfusion therapy** | **Occluder** | **Occluder size** | **Operation, complication, post-operation support** | **ICU stays** | **Alive** | **Cause of death** | **Survival Days** |
| --- | --- | --- | --- | --- | --- | --- | --- | --- | --- | --- | --- | --- | --- | --- | --- | --- | --- | --- |
| **1** | 31-May-12 | 45 | F | N | 5 | 28 | 23 | Apical | 10 | LAD | CABG | VSDO | 18 | Ventricular aneurysm resection, residual shunt, postoperative hemolysis |  | Y |  | 3146 |
| **2** | 10-Aug-12 | 71 | F | N | 4 | 82 | 78 | Apical | 7 | LAD | PCI | VSDO | 20 |  |  | Y |  | 3139 |
| **3** | 16-Nov-12 | 57 | M | N | 4 | 28 | 24 | Apical | 12 | LAD |  | VSDO | 22 |  |  | Y |  | 2990 |
| **4** | 4-Aug-13 | 68 | M | N | 3 | 30 | 27 | Apical | 6 | Triple vessel disease | PCI | VSDO | 16 | Residual shunt |  | Y |  | 2743 |
| **5** | 23-Aug-14 | 65 | F | N | 4 | 42 | 38 | Apical | 11 | - | - | VSDO | 18 | Residual shunt |  | Y |  | 2340 |
| **6** | 15-Jan-15 | 51 | M | N | 10 | 28 | 18 | Anterior | 8 | LAD | PCI | VSDO | 16 |  |  | Y |  | 2203 |
| **7** | 8-Jul-15 | 57 | M | N | 7 | 28 | 21 | Apical | 5 | LAD | PCI | VSDO | 18 | Residual shunt | 6 | Y |  | 2019 |
| **8** | 9-Mar-16 | 47 | M | Y | 4 | 14 | 10 | Apical | 10 | LAD | PCI | VSDO | 16 | CRRT | 15 | **N** | **LCOS, VF** | 16 |
| **9** | 1-Dec-16 | 64 | M | N | 10 | 160 | 150 | Anterior | 6 | LAD | PCI | VSDO | 16 | Residual shunt | - | Y |  | 1698 |
| **10** | 21-Dec-16 | 62 | M | N | 2 | 42 | 40 | Anterior | 14 | LAD | - | ASDO | 34 | Residual shunt | 1 | **N** | **LCOS** | 45 |
| **11** | 7-Mar-17 | 70 | F | N | 5 | 22 | 17 | Posterior | 10 | LAD | PCI | ASDO | 12 | Residual shunt | 27 | Y |  | 1410 |
| **12** | 19-May-17 | 75 | F | N | 2 | 14 | 12 | Apical | 11 | LAD | - | VSDO | 16 | Residual shunt | - | Y |  | 1342 |
| **13** | 28-Jun-17 | 67 | M | N | 1 | 107 | 106 | Posterior | 8 | LCX | PCI | VSDO | 18 | Residual shunt | - | Y |  | 1297 |
| **14** | 28-Sep-17 | 64 | F | N | 2 | 16 | 14 | Apical | 11 | - | - | VSDO | 24 |  | 11 | **N** | **LCOS, VF** | 18 |
| **15** | 6-Dec-17 | 60 | F | N | 2 | 27 | 25 | Apical | 3 | LAD | - | - | - | **Closure failure** | 23 | Y |  | 1142 |
| **16** | 24-Jan-18 | 53 | M | N | 7 | 24 | 17 | Anterior | 12 | LAD | - | VSDO | 26 | Residual shunt, IABP | 30 | Y |  | 1088 |
| **17** | 6-Jun-18 | 68 | F | N | 1 | 21 | 20 | Apical | 14 | LAD | - | VSDO | 16 | Residual shunt | 5 | Y |  | 960 |
| **18** | 13-Jun-18 | 67 | F | Y | 1 | 20 | 19 | Apical | 20 | LAD | PCI | VSDO | 28 | Postoperative hemolysis, | 3 | **N** | **MSOF** | 20 |
| **19** | 11-Jul-18 | 85 | M | Y | 2 | 14 | 12 | Apical | 6 | LAD | PCI | - | - | **Closure failure** | 3 | Y |  | 926 |
| **20** | 20-Jul-18 | 63 | F | N | 1 | 14 | 13 | Apical | 15 | LAD | - | VSDO | 28 | IABP, residual shunt | 19 | **N** | **LCOS** | 22 |
| **21** | 26-Jul-18 | 77 | F | N | 2 | 26 | 24 | Apical | 11 | LAD | - | VSDO | 24 | IABP | 19 | **N** | **LCOS** | 23 |
| **22** | 15-Aug-18 | 70 | M | N | 9 | 35 | 26 | Apical | 23 | LAD | PCI | ASDO | 28 | Residual shunt, postoperative hemolysis | - | Y |  | 892 |
| **23** | 7-Nov-18 | 60 | M | N | 3 | 21 | 18 | Apical | 18 | RCA |  | VSDO | 24 |  |  | Y |  | 802 |
| **24** | 15-Dec-18 | 63 | M | N | 5 | 56 | 51 | Apical | 5 | Triple vessel disease | PCI | VSDO | 16 |  |  | Y |  | 767 |
| **25** | 15-Dec-18 | 58 | M | Y | 1 | 14 | 13 | Anterior | 23 | RCA | PCI | ASDO | 34 | IABP | 19 | **N** | **LCOS** | 17 |
| **26** | 18-Dec-18 | 63 | M | N | 1 | 21 | 20 | Posterior | 10 | LAD | - | PADO | 18/20 |  | - | Y |  | 750 |
| **27** | 27-Dec-18 | 59 | M | N | 7 | 34 | 27 | Posterior | 12 | RCA | PCI | VSDO | 22 |  | 25 | Y |  | 758 |
| **28** | 10-Feb-19 | 82 | M | N | 4 | 14 | 10 | Anterior | 15 | LAD | CABG | VSDO | 22 | Tricuspid annuloplasty | 24 | Y |  | 697 |
| **29** | 13-Feb-19 | 62 | M | N | 1 | 17 | 16 | Apical | 10 | - | - | VSDO | 16 |  | 3 | Y |  | 704 |
| **30** | 6-Mar-19 | 62 | F | N | 8 | 21 | 13 | Posterior | 7 | RCA | PCI | VSDO | 24 | Residual shunt | 2 | Y |  | 688 |
| **31** | 8-Mar-19 | 70 | M | N | 1 | 23 | 22 | Apical | 7 | - | - | VSDO | 16 | Residual shunt, IABP, ECMO | 1 | **N** | **LCOS** | 23 |

AMI(acute myocardial infarction),VSR (ventricular septal rupture), TCC (Percutaneous transcatheter closure), M(male), F(female), LAD (left anterior descending coronary), RCA (right coronary artery), LCX (left circumflex coronary artery), PCI (percutaneous coronary intervention), CABG (coronary artery bypass grafting), Y (yes), N (no), VSDO (Ventricular septal defect occluder), ASDO (Atrial septal defect occluder), PDAO (Patent ductus arteriosus occluder), CRRT (continuous renal replacement therapy), IABP (intra-aortic balloon pump), ECMO (Extracorporeal membrane oxygenation), ICU (intensive care unit), LCOS (Low cardiac output syndrome),VF (Ventricular arrhythmia), MSOF (Multiple-system organ failure).Survival time were calculated from the day after VSR operation till the last follow-up (31-Dec-2020)

**Online Table 3: Characteristics of patients with VSR repair management according to operation timing**

|  | Operative management (n = 49) | Early repair operation (n = 18) | Delayed repair operation (n = 31) | *P* value |
| --- | --- | --- | --- | --- |
| Age, (years) | 63.7 ± 7.8 | 65.8 ± 8.85 | 62.4 ± 7.0 | 0.143 |
| Male sex, n (%) | 31 (63.3) | 12 (66.7) | 19 (61.3) | 0.767 |
| Cardiogenic shock, n (%) | 13 (26.5) | 6 (33.3) | 7 (22.6) | 0.508 |
| In-hospital stays, (days) | 28.0 [20.0 - 37.0] | 27.0 [18.5-37.3] | 28.0 [23.0-37.0] | 0.787 |
| AMI to VSR time, (days) | 3.7 ± 2.7 | 3.3 ± 2.3 | 3.9 ± 3.0 | 0.460 |
| VSR Type, n (%) |  |  | | 0.520 |
| Acute | 13 (26.5) | 4 (22.2) | 9 (29.0) |  |
| Subacute | 14 (28.6) | 7 (38.9) | 7 (22.6) |  |
| Late presentation | 22 (44.9) | 7 (38.9) | 15 (48.4) |  |
| Size of main VSR, (mm) | 12.7 ± 5.9 | 14.4 ± 6.3 | 11.6 ± 5.5 | 0.109 |
| Single VSR, n (%) | 43 (87.8) | 18 (100) | 25 (80.6) | 0.073 |
| VSR location, n (%) |  |  | | 0.914 |
| Apical | 31 (63.3) | 11 (61.1) | 20 (63.5) |  |
| Anterior | 9 (18.4) | 3 (16.7) | 6 (19.4) |  |
| Posterior | 9 (18.4) | 4 (22.2) | 5 (16.1) |  |
| VSR to operation time, (days) | 18.0 [13.0-24.5] | 12.0 [10.0-13.0] | 23.0 [19.0-27.0] | **< 0.001** |
| VSR repair management, n (%) |  |  | | 0.064 |
| Percutaneous TCC management | 31 (63.3) | 8 (44.4) | 23 (80.6) |  |
| Surgery management | 18 (36.7) | 10 (55.6) | 8 (25.8) |  |
| IABP support, n (%) | 13 (26.5) | 7 (38.9) | 6 (19.4) | 0.184 |
| Pre-repair | 5 (10.2) | 3 (16.7) | 2 (6.5) |  |
| Post-repair | 8 (16.3) | 4 (22.2) | 4 (12.9) |  |
| Post-repair ECMO support, n (%) | 2 (4.1) | 1 (5.6) | 1 (3.2) | 0.691 |
| Ventricular aneurysm, n (%) | 31 (63.3) | 9 (50.0) | 22 (71.0) | 0.219 |
| Ventricular arrhythmia, n (%) | 1 (2.0) | - | 1 (3.2) | - |
| Myocardial Infarction information |  | | | |
| STEMI, n (%) | 42 (85.7) | 16 (88.9) | 26 (83.9) | 0.701 |
| Infarct territory, n (%) |  |  | | 0.426 |
| Anterior | 39 (79.6) | 13 (72.2) | 26 (83.9) |  |
| Inferior | 9 (18.4) | 5 (27.8) | 4 (12.9) |  |
| Others | 1 (2.0) | - | 1 (3.2) |  |
| Prior Fibrinolysis therapy, n (%) | 5 (10.2) | 4 (22.2) | 1 (3.2) | 0.054 |
| CAG data, n (%) |  |  | | 0.526 |
| Negative | 4 (8.2) | 2 (11.1) | 2 (6.5) |  |
| LAD | 30 (61.2) | 9 (50.0) | 21 (67.7) |  |
| RCA | 7 (14.3) | 5 (27.8) | 2 (6.5) |  |
| LCX | 1 (2.0) | - | 1 (3.2) |  |
| Triple vessel disease | 4 (8.2) | 2 (11.1) | 2 (6.5) |  |
| Culprit artery treatment, n (%) |  | | | 0.734 |
| PCI | 19 (38.8) | 7 (38.9) | 12 (38.7) |  |
| CABG | 10 (20.4) | 4 (22.2) | 6 (19.4) |  |
| PCI+CABG | 3 (6.1) | 2 (11.1) | 1 (3.2) |  |
| Comorbidities, n (%) |  | | | |
| Current Smoker | 16 (32.7) | 5 (27.8) | 11 (35.5) | 0.754 |
| Current Drinker | 8 (16.3) | 2 (11.1) | 6 (19.4) | 0.693 |
| Hypertension | 27 (55.1) | 11 (61.1) | 16 (51.6) | 0.565 |
| Diabetes mellitus | 14 (28.6) | 4 (22.2) | 10 (32.3) | 0.527 |
| History of MI | 3 (6.1) | - | 3 (9.7) | 0.288 |
| History of stroke/TIA | 5 (10.2) | 3 (16.7) | 2 (6.5) | 0.342 |
| Hyperlipidemia | 5 (10.2) | 2 (11.1) | 3 (9.7) | 0.874 |
| Examinations |  | | | |
| Heart rate, (b.p.m.) | 92.5 ± 15.2 | 90.6 ± 16.8 | 93.6 ± 14.4 | 0.516 |
| SBP, (mmHg) | 110.6 ± 15.4 | 110.7 ± 17.3 | 110.6 ±14.5 | 0.985 |
| DBP, (mmHg) | 72.2 ± 10.4 | 68.9 ± 9.4 | 74.1 ± 10.6 | 0.089 |
| LVEF, (%) | 52.5 ± 9.1 | 53.6 ± 8.5 | 51.9 ± 9.5 | 0.538 |
| NT-pro BNP, (pg/mL) | 5339.0 [2651.0 - 9336.0] | 5590.0 [2688.5-10016.7] | 5160.0 [2547.5-8834.0] | 0.726 |
| CK-MB, (mmol/L) | 16.7 [10.8 - 24.6] | 23.7 [17.3-32.5] | 15.2 [9.5-20.3] | **< 0.001** |
| CTnI, (mmol/L) | 0.68 [0.16 - 4.61] | 4.1 [0.45-10.5] | 0.25 [0.10-2.66] | **< 0.001** |
| LDH, (U/L) | 579.0 [316.0 - 866.0] | 697.5 [427.8-1179.5] | 488.0 [279.0-742.0] | 0.135 |
| AST, (mmol/L) | 37.0 [20.0 - 88.3] | 40.0 [19.0-154.5] | 31.5 [20.3-58.5] | 0.574 |
| Hemoglobin, (g/L) | 128.1 ± 38.8 | 126.7 ± 33.1 | 128.9 ± 42.5 | 0.848 |
| WBC, (10^3^/μL) | 11.3 ± 5.49 | 13.3 ± 5.22 | 9.99 ± 5.36 | 0.045 |
| Creatinine, (µmol/L) | 88.4 [78.8 - 115.6] | 96.3 [76.8-137.4] | 87.4 [79.3-102.0] | 0.451 |
| Blood urea nitrogen, (mmol/L) | 10.4 ± 6.26 | 11.9 ± 8.56 | 9.48 ± 4.19 | 0.201 |
| eGFR, (mL/min/1.73 m^2^) | 83.1 ± 26.8 | 77.9 ± 32.2 | 87.6 ± 22.5 | 0.238 |
| Total bilirubin, (mmol/L) | 18.2 ± 13.3 | 20.6 ± 16.2 | 16.8 ± 11.3 | 0.335 |
| Albumin, (mmol/L) | 37.2 ± 7.33 | 38.4 ± 9.50 | 36.5 ± 5.70 | 0.390 |
| LDL-C, (mmol/L) | 2.34 ± 0.88 | 2.54 ± 0.81 | 2.18 ± 0.91 | 0.232 |
| 30-day mortality, n (%) | 9 (18.4) | 6 (33.3) | 3 (9.7) | **0.039** |
| Long-term mortality, n (%) | 12 (24.5) | 8 (44.4) | 4 (12.9) | **0.019** |

VSR (ventricular septal rupture), Percutaneous transcatheter closure (TCC), *VSR Type: Acute (Within 24 hours Post AMI), Subacute (Within 24-72 hours Post AMI), Late presentation (More than 72 hours Post AMI); SD (Standard Deviation), AMI (acute myocardial infarction), IABP (intra-aortic balloon pump), ECMO (extracorporeal membrane oxygenation), STEMI (ST segment elevate myocardial infarction), CAG (coronary angiography), LAD (left anterior descending), RCA (right coronary artery), LCX (left circumflex coronary artery), PCI (percutaneous coronary intervention), CABG (coronary artery bypass surgery),TIA (transient ischemic attack), SBP (systolic blood pressure), DBP (diastolic blood pressure), LVEF (left ventricular eject fraction), NT-pro BNP (N-terminal pro b-type natriuretic peptide), CK-MB(creatine kinase MB), CTNI (cardiac troponin I), LDH (lactate dehydrogenase), WBC (white blood cell), eGFR (estimated glomerular filtration rate), AST (aspartate transaminase), LDL-C (low-density lipoprotein cholesterol)
